# Correction: Water droplet on inclined dusty hydrophobic surface: influence of droplet volume on environmental dust particles removal

**DOI:** 10.1039/c9ra90018g

**Published:** 2019-03-05

**Authors:** Ghassan Hassan, Bekir Sami Yilbas, Abdullah Al-Sharafi, H. Al-Qahtani, Nasser Al-Aqeeli

**Affiliations:** Mechanical Engineering Department, King Fahd University of Petroleum & Minerals Dhahran Saudi Arabia bsyilbas@kfupm.edu.sa; Center of Excellence in Renewable Energy, King Fahd University of Petroleum & Minerals Dhahran Saudi Arabia

## Abstract

Correction for ‘Water droplet on inclined dusty hydrophobic surface: influence of droplet volume on environmental dust particles removal’ by Ghassan Abdelmagid *et al.*, *RSC Adv.*, 2019, **9**, 3582–3596.

The authors regret that the name of one of the authors (Ghassan Hassan) was shown incorrectly in the original article. The corrected author list is as shown above.

The Royal Society of Chemistry apologises for these errors and any consequent inconvenience to authors and readers.

## Supplementary Material

